# Anti-inflammatory effect of combining fish oil and evening primrose oil supplementation on breast cancer patients undergoing chemotherapy: a randomized placebo-controlled trial

**DOI:** 10.1038/s41598-023-28411-8

**Published:** 2023-04-20

**Authors:** Aleksandra Arsic, Predrag Krstic, Marija Paunovic, Jasmina Nedovic, Vladimir Jakovljevic, Vesna Vucic

**Affiliations:** 1 Group for Nutritional Biochemistry and Dietology, Institute for Medical Research, National Institute of Republic of Serbia, University of Belgrade, Belgrade, Serbia; 2 Department of Hematology, Military Medical Academy, Belgrade, Serbia; 3 Clinical Centre of Kragujevac, University of Kragujevac, Kragujevac, Serbia; 4 Department of Physiology, Faculty of Medical Sciences, University of Kragujevac, Kragujevac, Serbia

**Keywords:** Cancer, Medical research

## Abstract

Breast cancer is the most common malignant tumor and one of the leading causes of cancer-related death in women throughout the world. This study is a parallel, randomized, double-blind, controlled, 12-week supplementation trial, investigating the anti-inflammatory effects of dietary intake of fish oil and evening primrose oil (EPO), in patients with breast cancer undergoing chemotherapy. The primary outcomes were changes in the nutritional status and inflammatory cytokines of patients during the study. The secondary outcomes were changes in hematological and biochemical parameters and fatty acid profile. Of the 32 eligible patients, half of them is randomly assigned to a treatment arm with fish oil and EPO (n = 16), or a control arm (n = 16) with mineral oil as a placebo. The intervention group was taking 2 gel capsules of fish oil and 3 gel capsules of EPO (400 mg eicosapentaenoic acid, 600 mg docosahexaenoic acid, and 351 mg gamma-linolenic acid) fish oil and evening primrose oil for 12 weeks, during their chemotherapy. The control/placebo group was taking 5 gel capsules of 1g of mineral oil. One of the patients dropped out due to discontinuation of the treatment (in the placebo group) and two did not show up at the post-treatment measurements (in the intervention group), thus, 29 women completed the study. The results showed an increase in plasma levels of docosapentaenoic acid (22:5n-3), docosahexaenoic acid (22:6n-3), total n-3PUFA, vaccenic acid (18:1n-7), and a decrease in n-6/n-3 PUFA ratio in the intervention group. An increase in the plasma level of dihomo-gamma-linolenic acid (20:3n-6) was observed in the placebo group. There was no difference in plasma levels of interleukin (IL) IL-8, IL-10, and tumor necrosis factor-alpha, while the level of IL-6 decreased in both groups and was significantly lower in the intervention group at the end of the study. In conclusion, this supplementation improved the PUFA status and decreased the level of IL-6 in breast cancer patients undergoing chemotherapy. Consequently, this treatment may help reduce cancer complications resulting from impaired lipid metabolism and inflammation. ClinicalTrials.gov Identifier: NCT03516253. Date of registration 04/05/2018.

## Introduction

Breast cancer (BRC) is the most frequent cancer in women, with an incidence of 2.1 million new cases worldwide in 2020^1^. Adjuvant systemic therapies, such as chemotherapy, are still the golden standard in BRC therapy. In the past 35 years, the efficacy of chemotherapy has significantly increased, resulting in reduced morbidity and improved survival rate of cancer patients^2^. Despite the evident beneficial effects^3^, the treatments are related to complications and induce different side effects. Acute reversible side effects include alopecia, nausea, vomiting, fatigue, and myelosuppression, whereas long-term potentially irreversible side effects include neuropathy, cardiomyopathy, and acute leukemia^4^. Many of them are mediated by proinflammatory cytokines such as tumor necrosis factor-alpha (TNF-a) and interleukin 6 (IL-6)^5^, often found in high concentration in patients long-term after cancer treatment^6^. Additionally, chemotherapy significantly alters lipid metabolism, reducing the HDL-cholesterol and apoA1 and increasing apoB concentrations, as well as impairing nutritional status, leading to increased risk for the development of cardiovascular diseases^7,8^. Therefore, the routes for ameliorating side effects of chemotherapy with natural hypolipemic and anti-inflammatory agents during and/or after the treatment are increasingly the focus of numerous studies^9,10^.

The favorable effects of fish oil, rich in docosahexaenoic acid (22:6n-3, DHA) and eicosapentaenoic acid (20:5n-3, EPA) on clinical outcomes in patients receiving chemotherapy, have been documented in several studies^11^. EPA is the main precursor for the synthesis of anti-inflammatory cytokines. The consumption of EPA and DHA in BRC patients may lead to several beneficial outcomes, including maintenance of optimal body weight and nutritional status, reduction in proinflammatory cytokines, a decrease of fatigue and pain, improvement of the quality of life, as well as overall better recovery of the metastatic BRC patients^12,13^. Moreover, both in vitro and animal studies indicate the synergistic action of DHA and EPA with chemo- and radiotherapy in destroying tumor cells, reducing metastases, and inhibiting angiogenesis^14^. Regardless of these findings, there is no consensus on the dose and the duration of administration of fish oil, and n-3 polyunsaturated fatty acids (PUFA) are not a part of the multimodal therapeutic approach in BRC patients.

Apart from n-3 PUFA, levels of gamma-linolenic acid (GLA, 18:3n-6) in plasma lipids of BRC patients were also found to be extremely low, as compared with healthy individuals^15^. Although GLA belongs to n-6 PUFA, the latter mainly having pro-inflammatory potential, it exhibits anti-inflammatory properties through its conversion to dihommo-gamma-linolenic acid (DGLA) and its further conversion to anti-inflammatory prostaglandin PGE1^16^. As a rich source of GLA, evening primrose oil (EPO) alone or in combination with tamoxifen, exerted anti-cancer effects in BRC cell lines. Accordingly, it has been suggested that in combination with tamoxifen (endocrine therapy), EPO may improve the efficacy of therapy and reduce side effects in BRC patients^17^. However, there is a gap in knowledge on the potential benefit of EPO intake in BRC patients undergoing chemotherapy.

We hypothesized that fish oil combined with EPO may decrease systemic inflammation and improve the nutritional status and lipid metabolism of BRC patients undergoing chemotherapy. The benefit of simultaneous supplementation with anti-inflammatory n-3 PUFAs and GLA is supported by the results of our previous studies, showing low plasma levels of n-3 PUFA and GLA in BRC patients^15^, and on the beneficial effects of this combination in patients with rheumatoid arthritis^18^. Thus, the objective of the present study was to evaluate whether fish oil (rich in EPA and DHA), and evening primrose oil (rich in GLA) supplementation influence concentration of cytokine (IL-6, IL-8, IL-10, and TNF-alpha), nutritional status/body composition, and fatty acids profile in BRC patients receiving adjuvant chemotherapy.

## Results

GC analysis of fish oil and evening primrose oil capsules content is presented in Table 1. Results showed that fish oil contained 35.09% EPA and 22.68% DHA per tablet, and the rest were mixture of other PUFA, MUFA and SFA. EPO contained more than 80% linoleic and gamma-linolenic acids and the rest were MUFA and SFA. These results were expected and confirmed the label.

**Table 1:** Fatty acid profile in fish and evening primrose oil, Mean ± SD.

Fatty acids (%)	Fish oil	Evening primrose oil
Myristic acid (14:0)	2.63 ± 0.14	–
Pentadecylic acid (15:0)	0.23 ± 0.09	–
Palmitic acid (16:0)	6.26 ± 0.40	8.08 ± 0.08
Stearic acid (18:0)	2.61 ± 0.60	2.27 ± 0.08
Arachidic acid (20:0)	–	0.33 ± 0.02
Heneicosylic acid (21:0)	3.35 ± 0.46	–
Behenic acid (22:0)	0.35 ± 0.05	–
SFA	15.42 ± 0.26	10.67 ± 0.17
Pentadecenoic acid (15:1)	1.34 ± 0.22	–
Palmitoleic acid (16:1n-7)	3.22 ± 0.27	0.07 ± 0.01
Oleic acid (18:1n-9)	4.38 ± 0.24	6.87 ± 0.14
Vaccenic acid (18:1n-7)	–	0.82 ± 0.06
Eicosenoic acid (20:1n-9)	–	0.45 ± 0.04
MUFA	8.93 ± 0.25	8.21 ± 0.21
Linoleic acid (18:2n-6)	0.57 ± 0.15	**72.58 ± 0.22**
Gamma-linolenic acid (18:3n-6)	–	**8.54 ± 0.26**
Icosadienoic acid (20.2n-6)	0.89 ± 0.14	
Arachidonic acid (20:4 n-6)	2.62 ± 0.51	
Docosadienoic acid (22:2n-6)	7.85 ± 0.74	
n-6 PUFA	11.94 ± 0.06	81.12 ± 0.04
Eicosapentaenoic acid (20:5n-3)	**35.09 ± 0.15**	
Docosapentaenoic acid (22:5n-3)	5.94 ± 0.10	
Docosahexaenoic acid (22:6n-3)	**22.68 ± 0.50**	
n-3 PUFA	63.71 ± 0.45	

The most abundant fatty acids in oils are in bold.

SFA (saturated fatty acid), MUFA (monounsaturated fatty acid) PUFA (polyunsaturated fatty acid).

### Participant's characteristics

**Figure 1:**
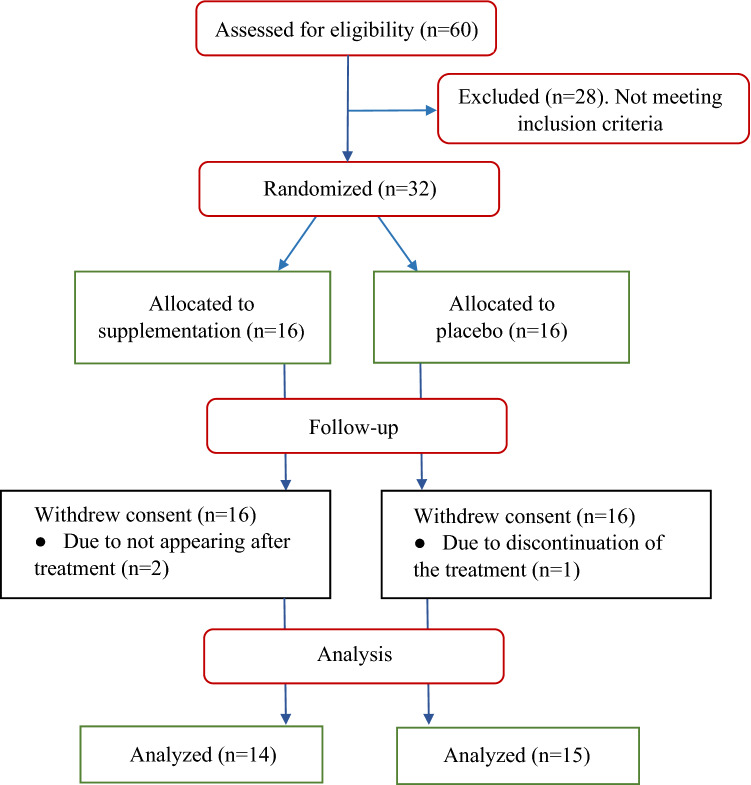
Disposition of the study participants.

Of the 60 patients screened for entry into the trial, 28 did not meet the criteria for randomization (Fig. 1). Of 32 eligible patients, 16 were randomly assigned to the intervention group and 16 to placebo. One patient dropped out due to discontinuation of the treatment (in the placebo group) and two did not show up at the post-treatment measurements (in the intervention group), thus, N = 29 women completed the study. As presented in Table 2 there were no inter- or intra- groups differences regarding examined nutritional status during the study.

**Table 2:** Characteristics and relevant parameters of the breast cancer patients on adjuvant chemotherapy at baseline and after 12 weeks (end of the study), Mean ± SD.

Parametes	Intervention Baseline (N = 14)	12 weeks (N = 14)	Placebo Baseline (N = 15)	12 weeks (N = 15)
Age	56.14 ± 7.47		60.40 ± 9.19	
Weight (kg)	71.78 ± 8.71	72.29 ± 8.92	79.81 ± 18.42	79.68 ± 16.98
Height (cm)	162.93 ± 6.68		163.55 ± 7.49	
Body mass index (BMI (kg/m^2^))	26.79 ± 5.30	27.19 ± 4.64	29.93 ± 6.43	29.69 ± 6.08
FFM (kg)	44.72 ± 4.64	45.72 ± 5.78	48.07 ± 6.88	48.41 ± 6.20
TBW (kg)	32.91 ± 3.39	33.17 ± 2.90	35.40 ± 5.11	35.63 ± 4.53
ICW (kg)	20.17 ± 2.11	20.31 ± 1.86	21.67 ± 3.09	21.81 ± 2.76
ECW (kg)	12.74 ± 1.31	12.86 ± 1.07	13.73 ± 2.07	13.82 ± 1.81
WHR	0.97 ± 0.07	0.97 ± 0.08	1.00 ± 0.08	0.99 ± 0.08
SMM (kg)	24.31 ± 2.76	24.48 ± 2.42	26.26 ± 4.02	26.47 ± 3.63
BFM (kg)	27.06 ± 10.15	27.29 ± 10.12	31.74 ± 14.00	31.27 ± 13.55
PFM (%)	36.76 ± 10.94	36.70 ± 10.80	38.43 ± 8.82	37.97 ± 8.79
Mineral (kg)	3.08 ± 0.34	3.07 ± 0.32	3.31 ± 0.48	3.33 ± 0.50
Protein (kg)	8.71 ± 0.92	8.78 ± 0.80	9.38 ± 1.34	9.43 ± 1.20
Stage of disease	Case/total		Case/total	
IIa	7/14F		6/15	
IIb	5/14		6/15	
IIIa	2/14		3/15	

FFM (Fat free mass), TBW (Total body water), ICW (intracellular water), ECW (extracellular water), WHR (waist-hip ratio), SMM (Skeletal muscle mass), BFM (Body fat mass), PBF (Percent body fat).

### Biochemical and hematological parameters in intervention and placebo groups

Table 3 presents values of hematological parameters, at baseline and after 12 weeks. The count of leukocytes and erythrocytes, and the level of hemoglobin significantly decreased throughout the intervention period in both groups, without inter-group variabilities. Regarding biochemical parameters, all remained stable in both groups during the study (Supplementary Table S1).

**Table 3:** Hematological parameters of the breast cancer patients on adjuvant chemotherapy at baseline and after 12 weeks (end of the study), Mean ± SD.

Blood count	Intervention group-start (N = 14)	Intervention group-the end (N = 14)	Placebo group-start (N = 15)	Placebo group-the end (N = 15)	p
Le (× 10^9^/l)	5.49 ± 2.08	3.26 ± 1.47***	6.49 ± 1.66	4.76 ± 1.73**	
Er (× 10^12^/l)	4.35 ± 0.42	3.92 ± 0.58*	4.68 ± 0.33	4.24 ± 0.33**	
Hb (g/l)	130.07 ± 11.80	117.57 ± 15.41**	137.33 ± 11.01	126.60 ± 9.19**	
Plt (× 10^9^/l)	249.57 ± 72.68	303.50 ± 148.69	264.33 ± 55.42	281.38 ± 110.40	

Leukocytes (Le), Erythrocytes (Er), Hemoglobin (Hb), Platelets (Plt). *p < 0.05, **p < 0.01, ***p < 0.001 indicates a statistically significant difference from the baseline for the same group. p values represent statistically different between the intervention and placebo groups after the treatment.

### Fatty acid profile and estimated activity of desaturase and elongase in intervention and placebo groups

The proportions of fatty acid changed in both groups. As expected, the supplementation with fish oil and EPO led to significant changes in FA profiles in the intervention group, resulting in increased plasma levels of n-3 PUFA (docosapentaenoic acid (22:5n-3), DHA, and total n-3PUFA), followed by a decreased palmitoleic and oleic acid as well as the n-6/n-3PUFA ratio (p < 0.01) (Table 4). Interestingly, no differences in GLA concentration were found, but the concentration of docosatetraenoic (DTA, 22:4n-6) acid, as the last product of GLA metabolism, was increased after the supplementation period. Some changes were also detected in the placebo group. Namely, the level of DGLA (20:3n-6) was significantly higher at the end of the study (Table 4).

**Table 4:** Plasma fatty acids profile from breast cancer patients on adjuvant chemotherapy at the start and the end of the study.

Fatty acids (%)	Intervention group-start (N = 14)	Intervention group-the end (N = 14)	Placebo group-start (N = 15)	Placebo group-the end (N = 15)	p
Palmitic acid (16:0)	27.17 ± 2.19	27.14 ± 2.21	27.51 ± 1.78	27.41 ± 1.16	
Stearic acid (18:0)	12.54 ± 0.78	12.60 ± 1.01	13.25 ± 2.46	13.34 ± 2.26	
SFA	39.71 ± 2.25	39.74 ± 2.25	40.26 ± 1.50	40.06 ± 1.38	
Palmitoleic acid (16:1 n-7)	1.17 ± 0.45	1.00 ± 0.35	1.28 ± 0.41	1.45 ± 0.61	0.017
Oleic acid (18:1 n-9)	12.35 ± 1.40	11.98 ± 1.63	13.25 ± 2.46	13.34 ± 2.26	0.019
Vaccenic acid (18:1 n-7)	1.43 ± 0.37	1.81 ± 0.33*	1.75 ± 0.48	1.77 ± 0.40	
MUFA	14.95 ± 1.82	14.80 ± 1.77	16.29 ± 3.01	16.57 ± 2.87	
Alpha-linolenic acid (18:3 n-3)	0.28 ± 0.25	0.21 ± 0.11	0.28 ± 0.15	0.22 ± 0.11	
Eicosapentaenoic acid (20:5 n-3)	0.53 ± 0.42	0.78 ± 0.48	0.54 ± 0.51	0.47 ± 0.45	
Docosapentaenoic acid (22:5 n-3)	0.48 ± 0.19	0.65 ± 0.22**	0.46 ± 0.15	0.47 ± 0.10	0.003
Docosahexaenoic acid (22:6 n-3)	2.61 ± 0.80	3.56 ± 0.81***	2.81 ± 0.92	2.59 ± 0.76	0.002
Total n-3PUFA	3.90 ± 1.44	5.20 ± 1.31***	4.10 ± 1.38	3.76 ± 1.22	0.011
Linoleic acid (18:2 n-6)	26.78 ± 4.88	25.62 ± 3.33	24.54 ± 3.39	23.91 ± 3.96	
Gamma-linolenic acid (18:3 n-6)	0.53 ± 0.16	0.50 ± 0.18	0.52 ± 0.19	0.56 ± 0.19	
Dihomo-gamma linolenic acid (20:3 n-6)	3.66 ± 0.77	3.45 ± 0.66	3.50 ± 1.13	3.98 ± 0.74*	0.008
Arachidonic acid (20:4 n-6)	10.05 ± 1.85	9.80 ± 2.89	10.16 ± 2.64	10.56 ± 2.66	
Docosatetraenoic acid (22:4 n-6)	0.42 ± 0.20	0.90 ± 0.64**	0.63 ± 0.49	0.60 ± 0.18	
Total n-6 PUFA	41.44 ± 3.09	40.27 ± 3.11	39.36 ± 2.58	39.61 ± 3.03	
Total PUFA	45.34 ± 2.69	45.46 ± 2.47	43.45 ± 3.03	43.38 ± 3.14	0.050
n-6/n-3	12.09 ± 4.95	8.22 ± 2.21**	10.60 ± 3.58	11.36 ± 3.13	0.020

The values are means ± SD. SFA-saturated fatty acid, MUFA-monounsaturated fatty acid, PUFA-polyunsaturated fatty acid. *p < 0.05, **p < 0.01, ***p < 0.001 indicates a statistically significant difference from the baseline for the same group. p values represent statistically different between intervention and placebo groups after the treatment.

Comparisons between groups at the end of the study, adjusted for baseline values, showed significantly higher levels, of DPA, DHA, n-3 PUFA, and total PUFA, in the intervention group. Additionally, lower levels of palmitoleic and oleic acids, DGLA, as well as an n-6/n-3 PUFA ratio (Table 4), as well as the lower estimated activity of SCD-16 were also noted in the intervention group after 12 weeks of treatment (Supplementary Table S2).

### Levels of cytokines in intervention and placebo groups

The concentrations of inflammatory markers IL-8, IL-10, and TNF-alpha remained unchanged, while the concentration of IL-6 significantly decreased in both groups during chemotherapy (Table 5). Additionally, the intergroup comparisons at the end of the study adjusted for baseline levels revealed a significantly (p < 0.01) lower concentration of IL-6 in the intervention group compared to the control group.

**Table 5:** The cytokines concentration in breast cancer patients on adjuvant chemotherapy at the start and the end of the study.

Parameters	Intervention group-start (N = 14)		Intervention group-the end (N = 14)		Placebo group- start (N = 15)		Placebo group- the end (N = 15)		p
	Median	Range	Median	Range	Median	Range	Median	Range	
IL-10 pg/ml	**7.26**	6.71–10.95	**7.07**	6.02–10.54	**7.26**	6.44–9.72	**7.15**	6.71–15.47	
IL-6 ng/ml	**30.67**	0.11–116.42	9.42***	0.95–44.23	**33.79**	0.95–70.08	12.76***	0.90–40.12	0.000
IL-8 pg/ml	**10.94**	9.21–16.21	**10.86**	7.08–34.31	**11.86**	9.45–19.66	**11.03**	9.31–18.45	
TNF-alpha ng/ml	**166.90**	69.30–307.88	**152.01**	74.52–198.12	**155.04**	86.70–204.37	**142.65**	98.14–131.91	

The most abundant fatty acids in oils are in bold.

Results are presented as median, upper and lower quartile with minimal and maximal values. ***p < 0.001 indicates a statistically significant difference from the baseline for the same group. p values represent statistically different between intervention and placebo groups after treatment.

IL, Interleukin; TNF, tumor necrosis factor.

## Discussion

This randomized double-blind controlled study investigated the effects of supplementation with an anti-inflammatory combination of n-3 and n-6 PUFA vs. placebo on body composition, lipid metabolism, and inflammatory response in breast cancer patients receiving the adjuvant chemotherapy during the period of 12 weeks.

Although chemotherapy commonly leads to changes in weight, BMI, waist circumference, and body fat percentage^19,20^, in this study there was no change in these parameters during the first 12 weeks of adjuvant chemotherapy in either of the groups. Freedman et al. have shown that although some women with BRC did not experience significant changes in weight or BMI after chemotherapy, they had unfavorable changes in body composition (decreased fat free mass and increased percentage of body fat)^21^. Although gains in weight and/or waist circumference are positively associated with baseline values of these parameters^22^, premenopausal women display a significantly higher risk for body fat percentage gains compared to postmenopausal women^20^, during chemotherapy. Maintenance of all anthropometric parameters and body composition in all participants of our study could probably be explained by the fact that only post-menopausal women were included. Moreover, body weight changes also depend on other variables such as the type and length of chemotherapy^23^, decreased physical fitness, anxiety, depression and appetite change^24^. Further, the literature data have shown the most prominent positive effects of supplements in cancer patients with cachexia^25^. However, all the patients included in this study had BRC at early stages, and the effects of supplements are not significant, but the follow-up of these patients could show if the nutritional intervention at early stages prevents nutritional decline in the future. This is important since it will show if the supplementation at early stages should be included in the BRC therapy.

Since leukopenia and neutropenia are common side effects of many cytotoxic agents^26^, often leading to delayed cycles of chemotherapy in cancer patients, we also followed changes in hematological parameters during this study. Although n-3 PUFA has a proliferative effect on lymphocytes^27^, and improves white and red blood cell counts in mice under chemotherapeutics^28^, only a few human studies showed a beneficial effect of PUFA supplementation on hematological parameters during chemotherapy^29^. In our study, fish oil and EPO supplementation did not prevent the reduction in hematological parameters during chemotherapy in BRC patients grade IIa–IIIa, which is in line with the results of other studies^29^.

Biochemical parameters were stable during the 12 weeks of the study in both groups. Although some other studies have shown unfavorable change in lipid profile during chemotherapy^7^, this was not a case in the present study.

The n-3 PUFA and GLA supplementation during chemotherapy in our study participants significantly increased DPA, DHA, total n-3 PUFA, and DTA levels, and decreased the n-6/n-3 PUFA ratio in the intervention group. The optimal n-6/n-3 PUFA ratio is important not only for reducing the BRC risk^30^ but also for decreasing the invasive potential of tumor cells^31,32^. Moreover, DHA can positively influence the effectiveness of chemo- and radiotherapy in BRC patients^33^. More precisely, DHA from food or supplements is quickly incorporated into all phospholipids, including those of tumor tissues, thus making the tissue more flexible and permeable to antitumor agents. Importantly, these supplements do not increase the susceptibility of healthy tissues to the drugs, e.g., to exposed toxicity of chemotherapeutics^33,34^. Therefore, the observed increase in DHA probably reflected the increase in DHA in the tumor tissue and could increase the effectiveness of chemotherapy in patients with BRC.

In the placebo group participants who remained on the standard dietary intake, only the DGLA concentration significantly increased after 12 weeks of the study, while it remained the same in the intervention group. DGLA is a precursor of arachidonic acid (AA), but also anti-inflammatory PGE1 and thromboxane A1, and could inhibit the synthesis of pro-inflammatory leukotrienes LTB4, LTC4, and LTD4. The higher concentrations of DGLA in the placebo group may indicate reduced conversion to AA and subsequent anti-inflammatory molecules. As the estimated activity of ∆5 desaturase, which catalyzes the conversion of DGLA to AA, did not change significantly during the study in both groups, the increased level of DGLA may be due to the chemotherapy-reduced conversion to anti-inflammatory molecules, further adversely affecting BRC patients and leading to inflammation.

Unlike the other members of the n-6 PUFA family, GLA exhibits anti-inflammatory, vasodilating, and anti-aggregation effects. In addition, GLA has a cytotoxic effect on tumor cells^35^, inhibits angiogenesis^36^, and stimulates apoptosis of breast, pancreatic, colon, and brain cancer cells^37,38^. In vitro studies have shown that GLA improves the effectiveness and reduces the side effects of antitumor therapy, and the response time to the therapy^39^. Despite the promising results, few interventional studies have addressed GLA supplementation in BRC. To the best of our knowledge, this is the first study that examined the effect of simultaneous supplementation with fish oil and EPO in women with breast cancer on adjuvant chemotherapy. In our intervention group the level of DTA (adrenic acid), as the final product of GLA conversion, was significantly increased after the supplementation, suggesting that DTA could inhibit AA release and formation of the LTB4^40^, leading to the decrease of inflammation in vivo. All the observed changes in PUFA have shifted the FA profiles in the intervention group towards anti-inflammatory profiles. It is worth to mention that although EPO contains 72% of linoleic acid (18:2 n-6), its effect in the supplemented group is not exerted, since the amount of LA in EPO is not significant in comparison to the dietary intake of this fatty acid.

Another beneficial effect of the supplementation, in this study, is decreased stearoyl CoA desaturase (SCD-16) activity and reduced levels of oleic and palmitoleic acid, since the carcinogenesis is closely related to the increased activity of enzymes involved in the biosynthesis of monounsaturated fatty acid (MUFA). Namely, the overexpression of SCD, which is responsible for the conversion of saturated fatty acids into MUFA, has been confirmed in HER2 + BRC^41^ and leads to increased MUFA, mostly oleic acid, in cell membranes. Oleic acid stimulates carcinogenesis by promoting the growth and migration of cells, especially those with high metastatic potential, as confirmed in HGC-27 gastric cancer and MDA-MB-231 BRC cell lines^42^.

In many inflammatory diseases, fish oil supplements are used as complementary therapy as they reduced the production of TNF-alpha, IL-1β, and IL-6 by mononuclear cells^43–45^. In this study, the IL-6 concentration dropped after 12 weeks of chemotherapy in both groups, but the decrease was much more prominent in the intervention group. This result demonstrates strong anti-inflammatory potential of the fish oil- EPO combination in BRC patients on chemotherapy. The changes in IL-10, IL-8, and TNF-a were not significant, possibly due to high inter-individual variabilities. However, we cannot rule out the possibility that there were modifications of these cytokines at local levels, e.g., in the tumor tissue, although they were not sufficient to modify the systemic levels. Similar findings have been reported in newly diagnosed BRC^46^. Nevertheless, this study has some limitations. First, this study was conducted at a single center, affecting generalizability. Second, the study population was relatively small.

In conclusion, supplementation with fish oil and EPO of BRC patients simultaneously with chemotherapy for 12 weeks, led to significant changes in fatty acid profiles of plasma lipids and lowered levels of IL-6, suggesting a beneficial effect on lipid metabolism and inflammatory response. Additional clinical trials with a higher number of participants and long-term supplementation are required to confirm the present results.

## Method

### Study population

This parallel, randomized double-blind placebo-controlled study included postmenopausal women with BRC, who have started adjuvant chemotherapy by anthracycline models. Of the 60 patients screened between February 2019 and May 2021 for entry into the trial, 28 did not meet the criteria for randomization. The patients (N = 32) between 45 and 70 years of age, were recruited, from the Military Medical Academy of Belgrade, Serbia and were equally randomly assigned to be given either EPO and FO (N = 16) or mineral oil (N = 16). One of the patients in the placebo group dropped out due to discontinuation of the treatment, and two did not show up at the post-treatment measurements (in the intervention group). A total of N = 29 patients remained in the study. Eligible patients were postmenopausal women with histopathological diagnosis of breast cancer, stage IIa-IIIa, with ER+ and/or PR+ breast cancer, and human epidermal growth factor receptor 2 (HER2) negative. Exclusion criteria were a metastatic disease, HER2+ cancer, previous stroke or heart attack, presence of significant neurological deficit and consciousness disorder, presence of other malignancies, thyroid disease, allergies to fish or EPO, use of statins, fish oil, and other supplements potentially influencing lipid metabolism. The study protocol was approved by the Ethical Committee of the hospital Military Medical Academy (MMA) and study was conducted according to the Declaration of Helsinki and the principles of Good Clinical Practice. Participants provided written informed consent at the time of recruitment. During the data analysis, the personal information of the patients was not identifiable. The present study is adhered to the CONSORT guidelines.

### Study design

This trial was a randomized 1:1 double-blind, placebo-controlled, parallel-group study conducted at the MMA and the Institute for Medical Research in Belgrade. The trial was registered at Clinical trials, NCT03516253 (Date of registration 04/05/2018). Random allocation was done by computer-generated permuted blocks of 4. The statistician who was not involved in the study generated a random allocation sequence, while the investigators enrolled participants, and assigned treatments. The patients were randomly assigned in a 1:1 to receive either supplements or a placebo. All capsules were identical in appearance, they were prepacked in bottles and consecutively numbered for each woman according to the randomization schedule. Patients and researchers were unaware of assignments to supplements and placebo groups until the statistical analysis was completed. The supplementation period started at the same time as chemotherapy. During 12 weeks of intervention, patients consumed two omega-3 Cardio capsules and three evening primrose oil capsules per day (Natural Wealth®, New York, USA), providing a total of 1000 mg EPA + DHA and 351 mg GLA. Compliance was assessed by the empty bottles that the participants returned at the end of the 12-week intervention period. Outcomes. The primary outcome was a change in nutritional status and level of interleukins in women with BRC during the study. The secondary outcomes were changes in hematological and biochemical parameters, fatty acid profile, in both groups during the study. Sample size. The sample size calculation was performed based on a study by Bougnox et al., which assessed the effect of DHA in BRC patients during chemotherapy^47^. Assuming a hypothesis that up to 5% of placebo group would have a positive response in fatty acid, inflammatory or nutritional parameters, we calculated that a minimum sample of 14 subjects in each group would allow the detection of differences caused by supplementation with n-3 PUFA, with a 80% power and 5% significance level.

### Anthropometry and biochemical assays

Anthropometric measurements (weight, height, and calculated BMI) were taken at baseline and at the end of the study, via standardized methods. Blood samples for all analyses were obtained in the morning, after an overnight fast, at the beginning and at the end of the intervention. Fasting serum glucose and lipids (triglycerides (TG), total cholesterol, high-density lipoprotein (HDL)-cholesterol low-density lipoprotein (LDL)-cholesterol) concentrations and hematological parameters were measured on the same day the samples were collected using clinical chemistry automated analyzer (Dimension RxL Max and ABX MICROS 60) and commercial Roche and ABX diagnostics kits, according to the manufacturer’s instruction. Blood collected in the tubes with EDTA was centrifuged to separate plasma and red blood cells and plasma was stored at − 80 °C until the determination of FA profiles.

### Analysis of the fatty acid profile of plasma lipids

Fatty acids from total plasma lipids were isolated as previously described^47,48^. Fatty acids were directly trans-esterified with 3 N HCl in methanol, at 85 °C for 60 min. Fatty acid methyl esters were analyzed by gas chromatograph SHIMADZU 2014 which was equipped with capillary column RESTEK Rtx 2330. The temperature program was 140–210 °C for 3°/min. Individual FA was identified by comparison with retention time of FA methyl esters commercial standards PUFA-2 (Supelco, Inc., Bellefonte, Pennsylvania, USA). Methyl esters from oils were prepared in the same way. Oils were analyzed in triplicate. The results are presented as a percentage of the total FA.

### Measurement of the plasma levels of cytokines

The cytokine concentrations in the plasma were determined using commercial ELISA assays (Human IL-6 DUO SET ELISA development kit and Human TNF-/TNFSF1A DUO SET ELISA development kit; R&D Systems, Minneapolis, Minnesota, USA) according to the manufacturer’s protocol. IL-8 and IL-10 were determined with ELABSCIENCE kits (Elabscience Biotechnology Inc Houston, Texas, USA) as per producer instructions.

### Statistical analysis

Statistical data analysis was performed by using SPSS 22.0 program. The Shapiro–Wilk test was used to check the distribution of variables. Descriptive statistics (mean, median, standard deviation, interquartile range) were used to describe examined parameters. Comparisons of values at the end of the study to baseline values were computed by two-factor analysis of variance for repeated measurements (2way RM-ANOVA) with an appropriate posthoc test. Time and treatment factors were analysed using ANCOVA where covariance is baseline values, Mann Whitney's U test and Wilcoxon's test were applied for non-parametric data. Significance was accepted at p < 0.05.

## Supplementary Information

Supplementary Tables.

## Data Availability

The datasets used and/or analyzed during the current study are available from the corresponding author on reasonable request.

## References

[1] World Health Organization (WHO). Global Health Estimates 2020: Deaths by Cause, Age, Sex, by Country and by Region, 2000-2019. Geneva, World Health Organization; 2020. https://www.who.int/data/gho/data/themes/mortality-and-global-health-estimates/ghe-leading-causes-of-death (2020). Accessed 11 December 2020.

[2] Falzone L, Salomone S, Libra M (2018). Evolution of cancer pharmacological treatments at the turn of the third millennium. Front. Pharmacol..

[3] Anampa J, Makower D, Sparano JA (2015). Progress in adjuvant chemotherapy for breast cancer: An overview. BMC Med..

[4] Mayer EL (2013). Early and late long-term effects of adjuvant chemotherapy. Am. Soc. Clin. Oncol. Educ. book. Am. Soc. Clin. Oncol. Annu. Meet..

[5] Sánchez-Lara K (2014). Effects of an oral nutritional supplement containing eicosapentaenoic acid on nutritional and clinical outcomes in patients with advanced non-small cell lung cancer: Randomised trial. Clin. Nutr..

[6] van der Willik KD (2018). Inflammation markers and cognitive performance in breast cancer survivors 20 years after completion of chemotherapy: A cohort study. Breast Cancer Res..

[7] Sharma M (2016). Chemotherapy agents alter plasma lipids in breast cancer patients and show differential effects on lipid metabolism genes in liver cells. PLoS ONE.

[8] Davoodi SH (2022). Oral propolis, nutritional status and quality of life with chemotherapy for breast cancer: A randomized, double-blind clinical trial. Nutr. Cancer.

[9] Aredes MA (2019). Efficacy of ω-3 supplementation on nutritional status, skeletal muscle, and chemoradiotherapy toxicity in cervical cancer patients: A randomized, triple-blind, clinical trial conducted in a middle-income country. Nutrition.

[10] D’Eliseo D, Velotti F (2016). Omega-3 fatty acids and cancer cell cytotoxicity: Implications for multi-targeted cancer therapy. J. Clin. Med..

[11] Chagas TR (2017). Oral fish oil positively influences nutritional-inflammatory risk in patients with haematological malignancies during chemotherapy with an impact on long-term survival: A randomised clinical trial. J. Hum. Nutr. Diet. Off. J. Br. Diet. Assoc..

[12] de Aguiar-Pastore-Silva J, Emilia-de-Souza-Fabre M, Waitzberg DL (2015). Omega-3 supplements for patients in chemotherapy and/or radiotherapy: A systematic review. Clin. Nutr..

[13] Trabal J, Leyes P, Forga M, Maurel J (2010). Potential usefulness of an EPA-enriched nutritional supplement on chemotherapy tolerability in cancer patients without overt malnutrition. Nutr. Hosp..

[14] Chapkin RS, McMurray DN, Lupton JR (2007). Colon cancer, fatty acids and anti-inflammatory compounds. Curr. Opin. Gastroenterol..

[15] Krstić P (2021). Similar fatty acid status of plasma lipids in postmenopausal women newly diagnosed with breast cancer and those receiving aromatase inhibitor therapy. Vojnosanit. Pregl..

[16] Dooper MMBW, Wassink L, M’Rabet L, Graus YMF (2002). The modulatory effects of prostaglandin-E on cytokine production by human peripheral blood mononuclear cells are independent of the prostaglandin subtype. Immunology.

[17] Abd-Alhaseeb MM, Massoud SM, Elsayed F, Omran GA, Salahuddin A (2022). Evening primrose oil enhances tamoxifen’s anticancer activity against breast cancer cells by inducing apoptosis, inhibiting angiogenesis, and arresting the cell cycle. Molecules.

[18] Vasiljevic D (2016). Evaluation of the effects of different supplementation on oxidative status in patients with rheumatoid arthritis. Clin. Rheumatol..

[19] Custódio IDD (2016). Impact of chemotherapy on diet and nutritional status of women with breast cancer: A prospective study. PLoS ONE.

[20] Fang Q, Gan L, Chen Y-Y, Shen K-W, Wu B-W (2018). Percent body fat change in chinese women after adjuvant chemotherapy for breast cancer. Med. Sci. Monit. Int. Med. J. Exp. Clin. Res..

[21] Freedman RJ (2004). Weight and body composition changes during and after adjuvant chemotherapy in women with breast cancer. J. Clin. Endocrinol. Metab..

[22] Harvie MN, Campbell IT, Baildam A, Howell A (2004). Energy balance in early breast cancer patients receiving adjuvant chemotherapy. Breast Cancer Res. Treat..

[23] van den Berg MMGA (2017). Weight change during chemotherapy in breast cancer patients: A meta-analysis. BMC Cancer.

[24] Lyon D (2015). Randomized sham controlled trial of cranial microcurrent stimulation for symptoms of depression, anxiety, pain, fatigue and sleep disturbances in women receiving chemotherapy for early-stage breast cancer. Springerplus.

[25] Silva JAP (2012). Fish oil supplement alters markers of inflammatory and nutritional status in colorectal cancer patients. Nutr. Cancer.

[26] Poikonen-Saksela P (2020). Leukocyte nadir as a predictive factor for efficacy of adjuvant chemotherapy in breast cancer. Results from the prospective trial SBG 2000–1. Acta Oncol..

[27] Calder PC, Yaqoob P, Thies F, Wallace FA, Miles EA (2002). Fatty acids and lymphocyte functions. Br. J. Nutr..

[28] Hardman WE, Moyer MP, Cameron IL (2002). Consumption of an omega-3 fatty acids product, INCELL AAFA, reduced side-effects of CPT-11 (irinotecan) in mice. Br. J. Cancer.

[29] Miyata H (2012). Randomized study of clinical effect of enteral nutrition support during neoadjuvant chemotherapy on chemotherapy-related toxicity in patients with esophageal cancer. Clin. Nutr..

[30] Yang B, Ren X-L, Fu Y-Q, Gao J-L, Li D (2014). Ratio of n-3/n-6 PUFAs and risk of breast cancer: A meta-analysis of 274135 adult females from 11 independent prospective studies. BMC Cancer.

[31] Xia S-H, Wang J, Kang JX (2005). Decreased n-6/n-3 fatty acid ratio reduces the invasive potential of human lung cancer cells by downregulation of cell adhesion/invasion-related genes. Carcinogenesis.

[32] Kobayashi N (2006). Effect of altering dietary omega-6/omega-3 fatty acid ratios on prostate cancer membrane composition, cyclooxygenase-2, and prostaglandin E2. Clin. Cancer Res. Off. J. Am. Assoc. Cancer Res..

[33] Bougnoux P, Hajjaji N, Maheo K, Couet C, Chevalier S (2010). Fatty acids and breast cancer: Sensitization to treatments and prevention of metastatic re-growth. Prog. Lipid Res..

[34] Molfino A (2017). Effect of oral docosahexaenoic acid (DHA) supplementation on DHA levels and omega-3 index in red blood cell membranes of breast cancer patients. Front. Physiol..

[35] Das UN (2006). Tumoricidal and anti-angiogenic actions of gamma-linolenic acid and its derivatives. Curr. Pharm. Biotechnol..

[36] Miyake JA, Benadiba M, Colquhoun A (2009). Gamma-linolenic acid inhibits both tumour cell cycle progression and angiogenesis in the orthotopic C6 glioma model through changes in VEGF, Flt1, ERK1/2, MMP2, cyclin D1, pRb, p53 and p27 protein expression. Lipids Health Dis..

[37] Menendez JA, Vellon L, Colomer R, Lupu R (2005). Effect of gamma-linolenic acid on the transcriptional activity of the Her-2/neu (erbB-2) oncogene. J. Natl. Cancer Inst..

[38] Kapoor R, Huang Y-S (2006). Gamma linolenic acid: An antiinflammatory omega-6 fatty acid. Curr. Pharm. Biotechnol..

[39] Kenny FS (2000). Gamma linolenic acid with tamoxifen as primary therapy in breast cancer. Int. J. cancer.

[40] Brouwers H (2020). Anti-inflammatory and proresolving effects of the omega-6 polyunsaturated fatty acid adrenic acid. J. Immunol..

[41] Vatten LJ, Bjerve KS, Andersen A, Jellum E (1993). Polyunsaturated fatty acids in serum phospholipids and risk of breast cancer: A case-control study from the Janus serum bank in Norway. Eur. J. Cancer.

[42] Li S (2014). High metastaticgastric and breast cancer cells consume oleic acid in an AMPK dependent manner. PLoS ONE.

[43] Baumann KH (1999). Dietary omega-3, omega-6, and omega-9 unsaturated fatty acids and growth factor and cytokine gene expression in unstimulated and stimulated monocytes. A randomized volunteer study. Arterioscler. Thromb. Vasc. Biol..

[44] Trebble T (2003). Inhibition of tumour necrosis factor-alpha and interleukin 6 production by mononuclear cells following dietary fish-oil supplementation in healthy men and response to antioxidant co-supplementation. Br. J. Nutr..

[45] Calder PC (2017). Omega-3 fatty acids and inflammatory processes: From molecules to man. Biochem. Soc. Trans..

[46] Paixão EMS (2017). The effects of EPA and DHA enriched fish oil on nutritional and immunological markers of treatment naïve breast cancer patients: A randomized double-blind controlled trial. Nutr. J..

[47] Bougnoux, P., Hajjaji, N., Ferrasson, M. N., Giraudeau, B., Couet, C. & Le Floch, O. Improving outcome of chemotherapy of metastatic breast cancer by docosahexaenoic acid: A phase II trial. *Br .J. Cancer* **101**(12), 1978–1985. (2009). 10.1038/sj.bjc.6605441. 10.1038/sj.bjc.6605441 PMC2779856 19920822

[48] Nikolic Turnic, T. *et al.* Hydroxymethylglutaryl coenzyme a reductase inhibitors differentially modulate plasma fatty acids in rats with diet-induced-hyperhomocysteinemia: Is ω-3 fatty acids supplementation necessary? *Front. Physiol.*10.3389/fphys.2019.00892 (2019). 10.3389/fphys.2019.00892 PMC6646860 31379600

