# Supplementary material for: Anti-inflammatory effect of combining fish oil and evening primrose oil supplementation on breast cancer patients undergoing chemotherapy: a randomized placebo-controlled trial
Source: Sci Rep. 2023 Apr 20;13:6449. doi: 10.1038/s41598-023-28411-8 (PMC10119093; doi:10.1038/s41598-023-28411-8)
Supplement: Supplementary file 1 — Supplementary Tables. [file 41598_2023_28411_MOESM1_ESM.docx]

Supplementary material

Table S1. Biochemical parameters of breast cancer patients on adjuvant chemotherapy at the start and the end of the study

| Biochemical  parameters | Intervention group-start (N=14) | Intervention group-the end (N=14) | Placebo group-start (N=15) | Placebo group the end (N=15) | p |
| --- | --- | --- | --- | --- | --- |
| Glucose (mmol/l) | 5.41±1.70 | 5.70±1.47 | 6.16±1.09 | 5.94±0.75 |  |
| Triglycerides (mmol/l) | 1.50±0.78 | 1.52±1.07 | 1.47±0.60 | 1.79±1.14 |  |
| Total cholesterol (mmol/l) | 5.56±1.02 | 6.47±2.87 | 5.46±1.28 | 5.63±1.39 |  |
| HDL-cholesterol  (mmol/l) | 1.40±0.29 | 1.41±0.29 | 1.55±0.37 | 1.47±0.26 |  |
| LDL-cholesterol (mmol/l) | 3.60±0.88 | 4.40±2.63 | 3.26±1.10 | 3.40±1.34 |  |
| Urea (mmol/l) | 4.57±2.08 | 4.34±1.52 | 5.11±1.83 | 4.89±1.21 |  |
| Creatinine  (mmol/l) | 67.71±8.28 | 69.79±12.84 | 66.13±12.39 | 64.93±10.45 |  |
| ALT (U/l) | 18.29±5.74 | 29.07±25.25 | 21.60±7.03 | 26.33±16.46 |  |
| AST (U/l) | 19.17±6.36 | 23.71±10.30 | 21.27±8.04 | 22.20±7.47 |  |

The values are means ±SD. p values represent statistically different between intervention and placebo groups after treatment. (HDL) high-density lipoprotein, (LDL)low-density lipoprotein, (AST) aspartate transaminase, (ALT) alanine transaminase

Table S2. Estimated activities of desaturases and elongase in breast cancer patients on adjuvant chemotherapy at the start and the end of the study

| Elongase and desaturase | Intervention group-start (N=14) | Intervention group-the end (N=14) | Placebo group-start (N=15) | Placebo group-the end (N=15) | p |
| --- | --- | --- | --- | --- | --- |
| SCD-16  (16:1n-7/16:0) | 0.04±0.02 | 0.04±0.01 | 0.05±0.02 | 0.05±0.02 | 0.016 |
| SCD-18  (18:1n-9/18:0) | 0.99±0.10 | 0.96±0.19 | 1.05±0.22 | 1.06±0.21 |  |
| Δ 6 desaturase  (18:3n-6/18:2 n-6) | 0.02±0.01 | 0.02±0.01 | 0.02±0.01 | 0.02±0.01 |  |
| Δ 5 desaturase  (20:4n-6/20:3n-6) | 2.83±0.62 | 2.95±1.03 | 3.40±1.83 | 2.82±1.15 |  |
| elongase (18:0/16:0) | 0.46±0.05 | 0.47±0.06 | 0.47±0.08 | 0.46±0.05 |  |

The values are means ±SD. p values represent statistically different between intervention and placebo groups after the treatment, (SCD) stearoyl- CoA-desaturase
